# Unraveling the Link Between Mitochondrial Dynamics and Neuroinflammation

**DOI:** 10.3389/fimmu.2021.624919

**Published:** 2021-03-16

**Authors:** Lilian Gomes de Oliveira, Yan de Souza Angelo, Antonio H. Iglesias, Jean Pierre Schatzmann Peron

**Affiliations:** ^1^ Neuroimmune Interactions Laboratory, Immunology Department – Institute of Biomedical Sciences (ICB) IV, University of São Paulo (USP), São Paulo, Brazil; ^2^ Neuroimmunology of Arboviruses Laboratory, Scientific Platform Pasteur-USP, University of São Paulo (USP), São Paulo, Brazil; ^3^ Loyola University Medical Center, Stritch School of Medicine, Loyola University Chicago, Chicago, IL, United States

**Keywords:** mitochondria, neuroinflammation, neurodegenerative diseases, Alzheimer disease, Parkinson disease, multiple sclerosis

## Abstract

Neuroinflammatory and neurodegenerative diseases are a major public health problem worldwide, especially with the increase of life-expectancy observed during the last decades. For many of these diseases, we still lack a full understanding of their etiology and pathophysiology. Nonetheless their association with mitochondrial dysfunction highlights this organelle as an important player during CNS homeostasis and disease. Markers of Parkinson (PD) and Alzheimer (AD) diseases are able to induce innate immune pathways induced by alterations in mitochondrial Ca^2+^ homeostasis leading to neuroinflammation. Additionally, exacerbated type I IFN responses triggered by mitochondrial DNA (mtDNA), failures in mitophagy, ER-mitochondria communication and mtROS production promote neurodegeneration. On the other hand, regulation of mitochondrial dynamics is essential for CNS health maintenance and leading to the induction of IL-10 and reduction of TNF-α secretion, increased cell viability and diminished cell injury in addition to reduced oxidative stress. Thus, although previously solely seen as power suppliers to organelles and molecular processes, it is now well established that mitochondria have many other important roles, including during immune responses. Here, we discuss the importance of these mitochondrial dynamics during neuroinflammation, and how they correlate either with the amelioration or worsening of CNS disease.

## Introduction

The central nervous system (CNS) depends on a complex and intricate network of molecular and cellular interactions to maintain appropriate function and homeostasis. This well-organized network when disturbed, leads to resident cells activation, inflammatory leukocyte infiltration, and further tissue damage. During recovery, counterregulatory mechanisms take place, and the activated cells return to the homeostatic state. However, in the absence of these finely tuned regulatory loops, the coordination is broken and chronic neurodegenerative and neuroinflammatory diseases may occur.

Neurodegenerative diseases represent a heterogeneous group of diseases of major public health concern. The World Health Organization (WHO) have estimated that until 2030, deaths attributed to neurological diseases will increased up to 12.22% (1). Due to their overly complex pathophysiology, interdisciplinary approaches and breakthrough science are highly needed to unravel disease mechanisms, and thus developing effective new therapies. In fact, with the extension of our life expectancy and the dramatic change in the age pyramid during the last decades, these studies are mandatory either to avoid or delay their impact on society and economy.

A common feature of neurodegenerative and neuroinflammatory diseases is the activation of CNS resident cells (2–4). Microglia and astrocytes may actively start, promote, or dampen neuroinflammation (5–8). The reason is because many immune-related receptors and molecules are extensively produced by these cells, not only during disease but also during physiological processes (3, 9). Conversely, these mechanisms demand high energy consumption, promoting important metabolic changes in the cell. In this context, the importance of mitochondria and mitochondria-related pathways is unquestionable.

More than just *power houses* of the cells (10), the role of mitochondria have been remarkably appreciated and revisited. Recent research has revealed important correlation of mitochondrial dynamics and the pathophysiology of brain diseases, as Alzheimer’s. Disease (AD), Parkinson’s Disease (PD) and Multiple Sclerosis (MS) (11–13). Disturbances in mitochondrial dynamics may influence many cellular and molecular pathways, as calcium-dependent immune activation, transcription factors phosphorylation, cytokine secretion, organelle transference and even cell death. Moreover, dysfunctional dynamics may also affect the release of mitochondrial damage-associated molecular patterns (mDAMPs), triggering innate immune responses in both resident and infiltrating cells (14). The release of mDAMPs leads to NOD-like receptors (NLRs), Toll-like receptors (TLRs) and cGAS-STING activation, promoting inflammatory cytokine, chemokines, and reactive oxygen species production, impacting disease outcome. However, although much has been learned regarding mitochondrial function during health and disease, mitochondrial dynamics during neuroinflammation and neurodegenerative disorders remains to be fully elucidated. Here, we aimed to summarize recent knowledge in the field, correlating dysfunctional mitochondrial dynamics with the worsening of CNS diseases.

## Mitochondrial Dynamics and Neuroinflammation

Mitochondrial dynamics is a process by which this organelle changes size, location, shape, and function inside the cell (15). Mitochondrial fusion and fission greatly correlate with metabolic changes, depending on the stimuli and energy demand, as it regulates cellular functions during health as well as during disease. There is now a better understanding of the changes that occur during mitochondrial dynamics changes and its relationship with CNS resident cells.

### Mitochondrial Location and Mitochondria-ER Communication

Mitochondria location within the cell is mostly regulated by the outer mitochondrial membrane (OMM), anchoring it to the cytoskeleton’s microtubules motor proteins, kinesin and dynein (16). For instance, mitochondria’s position in astrocytes influences Ca^2+^ levels, directly affecting astrocyte survival and communication with nearby neurons (17). Intracellular calcium level is dictated by the transferring between mitochondrial reticulum (mitRet) and endoplasmic reticulum (ER). Remarkably, several neurodegenerative diseases correlate with detrimental calcium homeostasis, evidenced by the disruption of mitRet and ER communication, as observed in Amyotrophic Lateral Sclerosis (ALS), a severe condition characterized by progressive weakness, muscle wasting and paralysis. Impaired electron transportation chain (ETC) and reduced glutamate uptake were already described in ALS. This greatly increases Ca^2+^ permeable activation of AMPA receptors, leading to excitotoxicity (16).

Mitochondria-ER associated membranes (MAMs) consist of around 1500 active proteins. Regardless of their fundamental importance for cellular metabolism and Ca^2+^ homeostasis, the molecular mechanisms that underly the recruitment and tethering of ER-mitochondria are not fully understood, and extensively debated. It has been proposed that MAMs tethering is dependent on the interaction between mitofusin 2 (MFN2) in the ER, and MFN1 and MFN2 in the OMM (16). Supporting this, ablation of MFN2 loosens ER–mitochondria interaction strongly impairing mitochondrial Ca^2+^ uptake (18). However, the role of MFN2 is not a consensus in the literature and some studies consider MFN2 a tethering antagonist that suppresses the excessive binding between the organelles, preventing toxic Ca^2+^ transfer within mit-Ret and ER (19, 20).

Tyrosine phosphatase-interacting protein 51 (PTPIP51), and the integral ER protein vesicle-associated membrane protein-associated protein B (VAPB) are also listed as tethering molecules for MAMs formation. Interestingly, during ALS, fronto-temporal dementia (FTD) and PD, disruption in the PTPIP51-VAPB interaction also induces dysregulated Ca^2+^ homeostasis and decreased ATP production (21, 22). Strikingly, it was orchestrated by fused in sarcoma (FUS) protein, and not by directly altering PTPIP51- VAPB expression, but by activating glycogen synthase kinase 3-beta (GSK-3b), evidencing a correlation between this molecule and ALS.

Additionally, the communication between mitochondrial voltage-dependent anion channel (VDAC) and inositol 1,4,5-trisphosphate receptor (IP3R) within the ER membrane *via* GRP75 was suggested as a bond of MAMs (23, 24). Curiously, the PD associated protein, DJ1, is necessary for mitochondrial Ca^2+^ uptake and was related with VDAC-IP3R-GRP75 complex in the maintenance of MAMS (25). Noteworthy the fact that mitochondrial Ca^2+^ uptake occurs in response to ER IP3R activation (19) and that fluctuations in channel activity does not affect the binding of MAM (26). Moreover, it is important to mention that IP3R activation is an important signaling pathway for immune response (27), as observed for nuclear factor of activated T cells (NFAT) (28).

NFAT activation typically leads to the transcription of inflammatory mediators that are upregulated during some neurodegenerative diseases. For instance, amyloid beta (Aβ) protein uptake by microglia induces dysregulated NFAT expression, increasing TNF-α secretion and neuronal death *in vitro* (29). Interestingly, Aβ and α-synuclein deposition, hallmarks of AD and PD, can both trigger inflammatory responses *via* TLR-2 and TLR-4, respectively (30–32). Conversely, Ma et al. (33) demonstrated that the crosstalk between TLR-4 and NFAT1 signaling into the mitochondria is a TRIF-dependent phenomenon, culminating in pro-inflammatory cytokine and ROS production, mitochondrial morphological changes and finally, prolonged microglia activation (33).

Corroborating the proinflammatory statement, it was demonstrated that cytokine activation of primary astrocytes and microglia upregulate intracellular Ca^2+^ mobilization and NFAT activation. NFAT upregulated genes are associated with a neurotoxic phenotype of astrocytes, known as A1 astrocytes. In A1 astrocytes (C3^+^GBP2^+^), NFAT is positively regulated by IL-1β and, in a positive feedback loop, IL-1β expression is NFAT and L-type Ca^2+^ channel dependent (34). Thus, bidirectional interactions between ER and mitochondrial Ca^2+^ levels, NFAT activation and upregulated inflammatory mediators, sustain a positive feedback loop that correlate with the chronicity of the neuroinflammatory microenvironment.

### Mitochondrial Dynamics and Programmed Cell Death

Besides location and interaction with other organelles within the cell, mitochondrial fusion and fission is a crucial process for regulating cell death. Fusion is coordinated by a family of GTPase proteins with tethering activity. This family of proteins is localized on the outer mitochondria membrane (OMM), highlighting MFN1 and MFN2, and in the inner mitochondria membrane (IMM), optic atrophy 1 (OPA1) (35). The steps that orchestrate mitochondrial elongation are not fully understood, but a model suggests that the interaction of MFNs from two opposing mitochondrion is stabilized by coil-coil heptad repeat-2 (HR2) (36), increasing the surface of contact (37). Following this, at the interaction site, guanosine triphosphate (GTP) is hydrolyzed culminating in conformational changes in the MFNs and thus, OMMs fusion. Different isoforms of OPA-1 such as long membrane-bound OPA1 (L-OPA1) and short soluble OPA-1(S-OPA1), generated by proteolytic cleavage of L-OPA1, are associated with fusion and fission balance (38). Complete fusion occurs when cardiolipin (CL) interacts with L-OPA1 resulting in IMM unification following OPA1-dependent GTP hydrolysis (39). Mitochondria elongation is associated with efficient metabolism and maintenance of ATP production even during nutrient deprivation, thus increasing cellular viability (40).

On the opposite, mitochondrial fission is initiated following the assembly of a pre-constriction site, directing the dynamin related protein 1 (DRP1) binding site of the OMM. One of the proteins that compose the pre-constriction site is fission protein 1 (FIS1), that also inhibits fusion by preventing GTP hydrolysis of OPA1 and MFN1/2 (41). The constriction site is not randomly located, instead is pinpointed on ER-mitochondria interaction site (42). ER tubules induce actin polymerization at the narrowing site, whereas myosin mediates actin contraction and mechanical pressure to ensure pre-constriction. Then, DRP1 is recruited forming a ring-like oligomer which following the GTP hydrolysis squeezes the pre-existing constriction site. Lastly, dynamin 2 is recruited to DRP1-compression site for mitochondrial fragmentation (43). The processes that coordinate the OMM fission are better known that the ones related to IMM. So far, it was shown that prior to DRP1 recruitment, Ca^2+^ promotes constriction in the IMM by favoring proteolytic cleavage of OPA-1 (44). Strikingly, the pre-constriction site is also spatially linked and critical to maintain mtDNA replication in the matrix (42). Fragmented mitochondria tend to present increased stress oxidation, membrane depolarization and impaired ATP production (45).

OPA1 and MFN2 genes are essentially related to the formation of healthy mitochondrial networks. Mechanistically, total or partial loss of function of OPA1 results in fragmented mitochondria, leading to a loss of mitochondria membrane potential (ΔΨm) and thus initiating autophagic and apoptotic pathways (46, 47). Importantly, in the CNS, these alterations may lead to massive neuronal and glial cell death, as seen in optic atrophy-1 and Charcot-Marie-Tooth disease and hereditary peripheral neuropathy (46, 47). Only recently, studies have described the role of mitochondrial fusion and fission in programmed cell death due to DRP1 and MFN2 interaction with BAX and BAK, respectively. As a result of exposure to toxic levels of nitric oxide (NO), BAX interacts with DRP1 in neurons inducing mitochondria fragmentation. In this context, inhibition of DRP1 impairs BAX deposition and pore formation, improving neuronal survival (48, 49).

The degradation of damaged organelles and cytosolic components usually results in autophagy, leading to the delivery of damaged cellular components to autophagosomes for degradation (50, 51). Mitochondria specialized autophagy, named mitophagy, is triggered by OPA-1, DRP1 and MFN2. These proteins, besides their regulatory role in mitochondria dynamics, are also responsible for autophagosome formation (50, 51). Mitophagy initiation is also dependent on PTEN−induced putative kinase 1 (PINK1) and E3-ubiquitin ligase protein (Parkin). These proteins accumulate in the OMM and ubiquitinate mitochondria target proteins (52, 53). As OMM has plenty of PINK1 and MFN2, Parkin is recruited from the cytoplasm and phosphorylated, hence exerting its ubiquitin activity (54). Consequently, DRP1, NF-κB essential modulator (NEMO) and mitochondrial Rho GTPase protein 1 (MIRO1) are targeted for proteasomal degradation (55, 56). Mitophagy is consolidated when MIRO1 is degraded by the proteasome and mitochondria is detached from its anchoring microtubules (57). Lastly, mitochondria is sequestrated in a double membrane vesicle that fuses with autolysosomes that further “digests” the organelle (58). Importantly, the correlation between Parkin and PINK1 mutations to autosomal-recessive cases of PD is widely known (59). In this context, the unbalance between damaged mitochondria and its removal, importantly contributes for PD progress. This unbalanced mitochondrial dynamic correlates with impaired clearance of dysfunctional organelles through Parkin and PINK1 pathway, resulting in deleterious accumulation. The benefits of mitophagy, however, goes beyond the removal of damaged mitochondria (59). Ip et al. (60) demonstrated that PINK1 is essential for microglial secretion of IL-10 and reduction of TNF-α secretion. Remarkably, elevated IL-10 secretion correlated with mitophagy induction in macrophages *via* mTORC1 inhibition and consequently decreasing inflammation (60). Using a mouse microglial cell line, it was shown that mitophagy increases ΔΨm and diminishes TNF-α induced apoptosis by hampering the increase in pro-apoptotic proteins (61). Furthermore, the role of mitophagy during neurodegenerative diseases overcome the regulation of immune responses. In a mouse model of AD, microglial cells under mitophagy have elevated levels of intracellular Aβ aggregates, suggesting increased phagocytic activity, and thus clearing the harmful Aβ deposits (62).

Autophagy may be beneficial to rebuild healthy mitochondrial dynamics after pro-inflammatory responses. Following mitochondrial fragmentation, autophagy is triggered due to dysregulated respiratory chain and ROS production. Such mitochondria alterations are promoted by IFN-γ and LPS upregulation of DRP1 and LC3, an autophagy-related protein. For instance, this mechanism is essential to restore tubular mitochondrial networks after inflammatory stimulation in astrocytes, as shown in a mouse model of cortical lesion. Interestingly, astrocytes located in the core or penumbra exhibited different mitochondrial patterns, with core mitochondria prominently fragmented, as opposed to those in the penumbra (63). This evidences the importance of the neuroinflammatory microenvironment in orchestrating mitochondrial shape and size.

### Astrocyte-to-Neurons Mitochondria Exchange

Many factors released from astrocytes provide neurotrophic and metabolic support for nearby neurons. These range from DNA, microRNAs, glucose-related molecules, cytokines and even organelles, such as the mitochondria (64, 65). Despite not completely understood, several reports have already demonstrated the importance of damaged and healthy mitochondria transference in between cells for neuronal metabolism and survival. During brain injury, astrocytes may release damaged mitochondria to minimize the amount of detrimental ROS and dysregulated Ca^2+^ balance (66). Conversely, healthy mitochondria may also be donated from astrocytes to damaged neighboring neurons, increasing its viability (67). Moreover, Davis et al. (68), firstly demonstrated that the exchange of mitochondria among neurons and astrocytes seem to work in a bidirectional way (68).

The release of mitochondria by astrocytes is a CD38/Ca^2+^ dependent phenomenon (69). It upregulates survival pathways in neurons after stroke, indicating a neuroprotective role for the glia-to-neuron mitochondria communication. Also, mitochondria acquired from astrocytes have a crucial role in maintaining neuronal energy production under glucose-oxygen starvation.

Joshi et al. (70) showed that previous mitochondria fragmentation is an essential step for organelle release to the extracellular space (70). They observed that inhibition of DRP1 diminishes astrocytic and microglial activation and ameliorates pro-inflammatory phenotype in mice models of AD, ALS and Huntington’s disease (HD). Interestingly, this phenotype was dependent on the release of damaged mitochondria by microglia cells, triggering neuronal death in consequence of A1-inflammatory-astrocyte activation (70).

## Mitochondrial DAMPs and Neurodegeneration

Since Polly Matzinger’s “danger theory” (71), the introduction of damage-associated molecular patterns (DAMPs) has greatly broadened our understanding of how the immune system works during tissue damage and repair (71). The idea of recognizing “danger” and “alarm” signals produced by cells, as DNA, ATP and HSPs (heat-shock proteins) (72), during inflammatory events, completely changed the view of inflammatory processes. Naturally, many advances in the biology of danger signals, along with the discovery of stress-associated molecules acting as DAMPs were achieved (71, 73). Accordingly, one important source of DAMPs that has gained increased attention is the *mitochondria*.

Examples of mDAMPs receptors are the classical PRRs (pattern recognition receptors), such as the TLRs, NLR (NOD-like receptors), as well as STING (stimulator of interferon-genes) and RAGE (receptor for advanced glycation products) (74). It is important to note that the signaling of these receptors, ultimately lead to inflammatory responses that may promote an auspicious environment for neurodegeneration. Moreover, the activation of TLR-7/9 and STING induces a IFN-I response (75) which has been recently demonstrated by microglial single-cell analysis in mice that, during aging, three clusters of interferon-responsive microglia appear, and that they correlate with subsequent CNS disease (76). Although the role for mitochondrial dynamics in this phenomenon is still to be addressed, the existence of mDAMP-IFN pathways denotes a possible correlation, as reviewed (74).

Cardiolipin is a phospholipid located at the IMM providing the structure for the electron transportation chain (ETC), binding to Complex IV and maintaining other ETC complexes and mitochondrial content in place and sharply functional (77). Cardiolipin molecules are particularly sensitive to oxidative damage created by unbalanced mitochondrial functioning and/or ROS produced by activated microglia. Interestingly, a highly oxidative environment causes loss of Δψm and promotes the repositioning of cardiolipin molecules to the OMM (77). Then, it can be sensed by cytosolic immune receptors, as NLRP3, initiating inflammasome activation, inflammatory cytokine secretion and dysregulation of mitochondrial dynamics (78). Other effects include loss of functional ETC and release of intrinsic apoptotic molecules located between the IMM and OMM, such as cytochrome C and SMAC/Diablo (79, 80). The release of Mitochondrial Transcription Factor A (MTFA) can also initiate inflammatory processes when released extracellularly. This is because MTFA shares high homology with High Mobility Group Box (HMGB), an important DAMP, and thus proinflammatory (65). Interestingly, a subset of Gamma-delta T cells (Tγδ) increased in Multiple Sclerosis (MS) patients has been shown to be activated by cardiolipin. Although their exact role is still not clear, it suggests an important role for cardiolipin also activating adaptive immune responses during CNS disease progression (81).

Mitochondrial DNA is the most studied mDAMP, and it has a high correlation with many pathological processes. Among their distinct characteristics, mtDNA codes only 13 proteins, including mitochondrial ribosomal subunits and ETC components, essential for proper mitochondrial function (82, 83). Failures in mitochondrial dynamics often result in the accumulation of mutated mtDNA, as they lack a robust repair mechanism (83, 84). This affects the cellular capacity in producing energy and also set in motion inflammatory processes (83, 84). mtDNA is not packed and has motifs usually perceived as harmful by innate immunity receptors. During mild stressful situations when apoptotic caspases are not mobilized, loss of mitochondrial membrane action potential (Δψm) for example, facilitates both OMM and IMM permeabilization and the induction of BAX/BAK pores, enabling mtDNA release to the cytosol. Then, it can be sensed by the cyclic GMP-AMP synthase (cGAS) receptor, activating its adaptor protein STING (stimulator of interferon genes) (85). Interestingly, this mtDNA-dependent Type I interferon (IFN-I) induction is beneficial in the context of viral infection because it primes the cell into an antiviral state (86). Still, the same issue occurs when mtDNA is present extracellularly, as internalized mtDNA signals through endosomal TLR-9, resulting in NF-κB and IRFs activation, and further interferon transcription (87, 88).

Another important mDAMP are mtROS produced at high levels by the mitochondria (89). Mitochondrial ROS (mtROS) are mainly byproducts of the mitochondrial ETC. During respiration, O_2_ that does not get reduced into H_2_O forms the $O_{2}^{-}$ radical specially by the activity of ETC complexes I and III, which can be later converted in H_2_O_2_ mainly by enzymes that are present in the organelle (90, 91). Other mitochondrial and cellular events can also enhance the production of mtROS, such as a decreased ΔΨm, inhibition of the ETC, mitochondrial Ca^2+^ influx, oxygen concentration and even mitochondrial morphology (90, 92). These molecules can act both as signaling (93) or as damaging molecules, causing mtDNA mutations, oxidation in fatty acids and amino acid residues, leading to deleterious disruption of the cellular redox signaling (94–96). Specially in the brain, the damage caused by ROS are linked to protein aggregation (93, 97).

In summary, although the mitochondria represent a vital organelle, providing energy and regulating metabolic processes of the cell, it also represents a “time-bomb” capable of inflicting and propagating devastating damage to the organism. This characteristic is especially significant on the CNS, where most resident cells are extremely susceptible to mitochondrial dysfunctions, as evidenced by the metabolic linked pathogeny of the neurodegenerative diseases, that will be further described in this review.

### UnDAMPening Mitochondria in Neurodegenerative Diseases

#### Mitochondria and Type I Interferon Responses

As mentioned, mDAMPs may signal through PRRs resulting in type I interferon responses (75, 98). Although their importance is mostly known during viral infections (99, 100), IFN-I responses in the CNS have a dual effect. In fact, there are evidences showing that IFN-I responses linked to mDAMPs in neurodegenerative diseases may also have a neuroprotective role (101, 102). Type IFN-I responses in the CNS have already been greatly reviewed by Deczkowska and colleagues (103), in which they discuss this complex signaling network duality. As an example, studies demonstrating that T cells derived IFN-I and IFN-γ are crucial to the blood brain barrier (BBB) permeability, as well as to the production of neurotrophic factors that aid the maintenance of cognitive functions. Corroborating this, the lack of IFN-β in the brain of knockout mice promotes progressive cognitive loss and impaired motor function (104).

Recognized mainly by TLR-9 and STING (75, 105), mtDNA has already been shown to be elevated in the serum of patients with ischemic brain injury (106, 107). Type I IFNs signaling rely on the interaction with Type I Interferon Receptors (IFNAR) and subsequent intracellular cascades that culminates in the phosphorylation of Jak-STATS and IRFs, and the transcription of Interferon Stimulated Genes (ISGs) (100). IFNAR are present in many cell types and, recently, transcriptome analysis in the brain showed the expression of IFNAR1 and IFNAR2 in almost every brain cell subset, including glial cells and neurons (108). In the CNS, the major IFN-αβ secreting sources are astrocytes and microglia. However, during neuroinflammation, disruption of BBB integrity facilitates the infiltration of IFN-I secreting cells, as monocytes and neutrophils (103, 108–111). Moreover, it is important to mention that the concept of CNS immune privilege is being extensively revisited, as many cell types are being described in the brain-circulation interfaces, as the choroid plexus and the meninges (112, 113), as well as within a complex network of lymphatic vessels (114).

It is also known that autocrine action of IFN-I induces significant shifts of the cellular metabolism, as augmented fatty acid oxidation and OXPHOS (65, 115, 116). These shifts are particularly important to astrocytes, as they rely on a tight controlled metabolite production to energetically supply nearby neurons, mainly by the lactate shuttle. For example, unbalanced glycolysis lowers the expression of glutathione, facilitating oxidative damage (65). Zhang et. al. 2019 recently reported that lactate, a key metabolite of the glycolytic pathway, interacts with the Mitochondrial Antiviral-Signaling protein (MAVS), preventing its oligomerization and maintaining Hexokinase 2 activity (HK2) (117). Although this was described using a model of viral infection, MAVS is increasingly showing to interact with many important enzymes beyond its role as an adaptor for cytosolic PRRs RIG-I and MDA-5. Importantly, the signaling trough MDA-5/MAVS has shown to be one of the main recognition pathways of cytosolic mtDNA, as evidenced during BAK/BAX mitochondrial disruption and failures of mtDNA turnover, being responsible for the major mtDNA induced IFN-I response (118, 119). Also, recent reports evidenced that phospholipase A2 binds to MAVS causing a disruption in the HK2 activity and increasing NF-κB phosphorylation, a novel pathway described in the experimental autoimmune encephalomyelitis (EAE) model (120). This not only give us important cues on how mtDAMPs and dysregulated type I IFN responses could accentuate neurodegenerative diseases, but also brings us new therapeutic avenues.

### Mitochondria as Borrowers: Sphingolipid Metabolism and Demyelination Processes

As discussed earlier, the metabolism of sphingolipids is an important player in neuroinflammation, as they critically participate in myelin maintenance (121, 122). For example, the compromised action potential of neurons, that is caused by intracellular changes or by the progressive loss of myelin due to metabolic failure of oligodendrocytes (123), clearly evidences the importance of a proper mitochondrial functioning (124).

The myelination process is tightly regulated both during neurodevelopment and tissue repair, when oligodendrocytes keep contributing for myelin remodeling (125–127) and remyelination (128). It requires great amount of energy, leading to high oxygen and ATP consumption, evidenced by high mitochondrial content within oligodendrocyte’s interface with myelin sheets (129). Thus, oligodendrocytes support the long-term myelination by maintaining high glycolytic rates. Conversely, mtDNA mutations in mitochondrial complex IV (mCOX-IV) subunits correlate with more extensive demyelination (129, 130). This phenomenon, along with increased iron deposits in oligodendrocytes, lowers the expression of anti-oxidative enzymes, rendering this cell population exceptionally susceptible to oxidative damage (125, 131), facilitating disease progression.

These characteristics are most evident in diseases as MS and AD, where the inflammatory milieu may drastically affect oligodendrocytes. Cytokines as IL-1, TNF-α and IFN-γ, may cause important mitochondrial distress. In fact, IFN-I impair glycolysis in oligodendrocytes, which is crucial for maintaining axonal integrity through myelin remodeling (132–134).

Furthermore, myelin production itself can be targeted during CNS pathologies. The sphingomyelinase/ceramide pathways play important roles in oligodendrocyte death by promoting the release of ceramide. Ceramide is the precursor of sphingomyelin lipids, the main component of myelin (135). This molecule, like other sphingolipids, has important bioactive functions, as promoting apoptosis and cell cycle arrest (11, 135). Increasing evidences has shown that ceramide can act directly on mitochondria (122) and also activate the NLRP3 inflammasome (136). In rat liver, it has been demonstrated that ceramide can be found in intimate contact with the IMM and OMM, thus leading to loss of Δψm and activating intrinsic apoptotic pathways and mitochondrial dynamics disbalance (137). This has also been discussed during CNS inflammation, as inflammasome activation by ceramide leads to hyperphosphorylation of leptin receptor (Obr) and thus abrogating signaling pathway, as observed during obesity and metabolic syndrome (135, 138).

### A STING in the Brain

Although the loss of Δψm is mostly studied during mitochondrial distress, novel data evidence that this event is also crucial for activating cytokine signaling cascades. For instance, the consequent Δψm mediated release of mtDNA can trigger cGAS activation (102, 139–141). Recent structural analysis revealed insights on how cGAS senses different dsDNA residues and, interestingly, it has a higher preference for mtDNA (142).

Discovered in the last decade (143), the cGAS-STING pathway has an important role during intracellular infections, being only recently valued under different contexts. CGAS catalyzes the production of cGAMP (cyclic GTP-AMP) in the presence of cytosolic dsDNA, serving as a second messenger for the activation of the STING adaptor protein. This promotes the phosphorylation of the Tank Binding Kinase 1 (TBK1) protein and further IRF3 nuclear translocation and IFN I transcription (144). Of note, the STING induction of type I IFN responses is a process that occurs only when the cell are not mobilizing apoptotic caspases (85). Thus, it evidences the importance of the cGAS-STING-IRF3 axis during neuropathology, as traumatic brain injury and hypoxia (145–147).

cGAS/STING also initiates NLRP3 responses by the elevation of K^+^ influx post lysosomal rupture (148). The NLRP3 induced IL-1β is an important acute phase cytokine that is critical to the pathophysiology of CNS diseases, as AD, PD, MS and even seizure disorders (72, 149–151). Recent research have shown that altered Δψm is dependent on IL-1R activation for further NF-κB, IRF3 and IFN-I expression (150). This novel pathway induces the release of mtDNA and further cytosolic detection by cGAS, but it is important to note that this discovery was made in monocytes and transformed lung cells (150). However, evidences indicate the presence of this axis in the CNS, as STING also modulates microglial reactivity during EAE (98, 152, 153). Moreover, the antiviral drug Ganciclovir promotes beneficial STING dependent type I IFN response in EAE model, dampening the harmful activity of activated microglia (154). Interestingly, mice knocked-out for mitophagy genes, as Parkin and PINK, that leads to inflammation and neuronal death in PD, had more prominent loss of dopaminergic neurons, which was reverted in the absence of STING. This provides an important link between STING and PD pathogenesis, evidencing the need for more studies on the biology cGAS-STING during neurodegenerative and neuroinflammatory diseases (155).

### Greasing Brain Engines: Cardiolipin

Damage to the mitochondrial membrane accounts for the release of mDAMPs. Accordingly, cardiolipin holds great responsibility for the structural stability of the ETC and the functional mitochondrial shape (156, 157). Cardiolipin is mostly associated with heart diseases, mainly due to its high content and impact in this organ (130). On the other hand, its impact over the CNS is an issue that has recently gained attention. Mutations in genes involved in cardiolipin biogenesis, *e.g.* the trans-acylase tafazzin (TAZ), have shown to affect cognitive functions in TAZ knock-out mice, expanding their classical role in X-linked myopathies (12, 158). This lipid is found in the body in different isoforms and, despite the dominance of the tetra linoleoyl cardiolipin isoform in the periphery, studies showed that in the CNS, there is a huge number of cardiolipin isoforms, distributed among different brain regions and cellular subtypes (159). Differences in cardiolipin composition and isoforms correlate to mitochondrial position inside the cell. For instance, as total cardiolipin increases, the closer they are to the synapses (160), which seem to correlate with the capacity of cardiolipin to influence ATP production (161).

Unsaturated lipids as cardiolipin are affected by mtROS and have their function compromised during oxidative stress (162). Proportional to the extension of cardiolipin peroxidation, there is a massive reduction of mitochondrial production of ATP due to Δψm loss and impairment of ETC complexes I, III, IV and V. Conversely, impaired ATP production by CNS cells is a common factor in aging and degenerative diseases (84). Studies in PD evidenced that cardiolipin interacts with α-synuclein by modifying its structure and exerting a protective role, by preventing its aggregation. Regarding this issue, divergent results demonstrated that this interaction can also affect cardiolipin functioning, resulting in increased pathology (163, 164). Moreover, α-synuclein binding to cardiolipin impairs the detection of cytosolic cytochrome c and thus inhibiting apoptotic cell death by dampening cellular oxidative stress (165).

The presence of cardiolipin in the OMM induces the recognition and further engulfment of dysfunctional mitochondria by LC3 mediated autophagy. Under conditions when the number of dysfunctional mitochondria exceeds the autophagy capacity, cardiolipin recruits BAX to form pores that release cytochrome c to the cytoplasm and triggering apoptosis. Thus, apart from the ETC complexes, cardiolipin also anchors important kinases that participate in the translocation of lipidic content through the mitochondrial membrane. When this mechanisms are impaired, it leads to a deleterious accumulation of dysfunctional mitochondria (156, 166).

### Give Me Fuel, Give Me Fire: Inflammasome Activation and Neuroinflammation

In addition to the induction of type I IFN response, mDAMPs also modulate the activation of inflammasomes. Canonical inflammasome activation leads to the proteolytic cleavage of pro-caspase-1 to caspase-1 and the subsequent pro-IL-1β/IL-18 and gasdermin 1 for further extracellular release (167). Both cytokines strongly activate pro-inflammatory responses, and they have an unquestionable importance during neuroinflammation, for instance, promoting the disruption of the blood-brain-barrier (BBB) and ROS production.

NLRP3 inflammasome is activated by a wide range of molecules, including mtROS, cardiolipin and mtDNA (148, 167). Importantly, for NLRP3 complete activation, an initial priming step is required to increase the expression of inflammasome effector molecules, as the NLRP3 itself, caspase-1 and pro-IL-1β. This is mediated by the activation of TLR-4, NOD receptor 2 (NOD2) and cytokines as TNF-α and IL-1β itself. This leads to NF-κB phosphorylation and nuclear translocation to promote NLRP3-related gene transcription (167–169). However, how inflammasome senses and interacts with stressors and the details of its activation remains not fully understood.

Interestingly, inflammasome activation is closely related to ER and mitochondria communication in many ways, and mitochondrial Ca^2+^ imbalance may also result in inflammasome activation (170). This may occur either by directly promoting NLRP3 complex formation or by mitochondrial Ca^2+^ overload and further mitochondrial dysfunction. Corroborating this, blocking ER IP3R, a major regulator of ER-to-cytoplasm Ca^2+^exchange, effectively attenuates NLRP3 activation (171, 172). Accordingly, mitochondria calcium homeostasis is closely related to ER Ca^2+^ metabolism since the MAMs plays a key role in material transfer and signaling between both organelles (16).

Inactivated forms of NLRP3 are localized in the ER membrane, although under certain stimuli, NLRP3 is redistributed across the MAMs (173). Under stress conditions, cardiolipin is exposed on the OMM and serves as a bridge between NLRP3 and MAMs (78). The localization of NLRP3 over the MAMs induces clusters of mitochondria around the Golgi apparatus and the release of NLRP3 to the cytosol for inflammasome mature form assembling (173). The mitochondrial location of NLRP3 is also affected by the interaction between MFN2 and MAVS during viral infections, which recruits the inflammasome to the MAMs. However, MAVS are not essential for NLRP3 activation under other stimuli (16). Mitophagy is also an important player during inflammasome activation since the removal of impaired mitochondria reduces ROS production. The continuous production of ROS occurs during oxidative phosphorylation and several studies have demonstrated that inhibitors of complex I, II and III develop an important role in mtROS production reflecting in decreased inflammasome activation (174, 175).

Interestingly, mtROS and Ca^2+^ have a synergic role for pore formation within mitochondria membranes. Mitochondrial permeability transition (MPT) pores allow the release of mtDNA. Interestingly, oxidized mtDNA in the cytoplasm triggers IL-1β secretion by preferentially activating NLRP3 but not AIM-2 (176). Additionally, IL-1β production was significantly enhanced with oxidized versus normal DNA (176). Importantly, the induction of NLRP3 also induces mtDNA release in the cytosol, thus creating a positive loop in the induction of inflammasome pathway (14, 176).

CNS disorders may occur due to NLRP3 dysfunctions and its close link with mitochondrial health (177). In EAE for example, increased levels of IL-1β and NLRP3 were related to disease progression. Additionally, microglia deletion of A20, an immune suppressive protein correlated with increased NLRP3 activation and IL-1β/IL18 secretion (178). Conversely, the role of IFN-β, a well-established treatment for MS, was demonstrated to be dependent on NLRP3 activation during EAE (179).

The correlation of inflammasomes and AD pathophysiology is also debated, since increased levels of IL-1β were reported in the Aβ neighboring microglia cells. Halle et al. (180) observed that Aβ phagocytosed by microglia triggers caspase-1 and subsequent release of IL-1β, *in vitro*. Corroborating this, *in vivo* studies shown that loss of NLRP3 is associated with reduced Aβ deposition, cytokine production and lead to ameliorated spatial memory deficits in AD mouse model. As Aβ, α-synuclein also induces NLRP3 activation in mouse microglial cell line. *In vivo*, the administration of caspase-1 inhibitor decreases the activation of NLRP3 and induces an increase in the number of dopaminergic neurons, consequently relating a better PD prognosis (177). Interestingly, caspase-1 is also able to cleave α-synuclein, and NLRP3 inhibition abrogates synuclein aggregation, ameliorating cell damage in murine PD model (181).

Penghu and collaborators showed that mtROS induced NLRP3 activation in hippocampal microglia (182). Sarkar et al. (182) evidenced that the inhibition of mitochondrial complex I by rotenone increased ROS production, leading to augmented cleavage of caspase-1 and NLRP3 expression in microglia cells. Conversely, they also evidenced that this pathway culminates in a more prominent dopaminergic neuronal loss (183).

## Concluding Remarks

**Figure 1:**
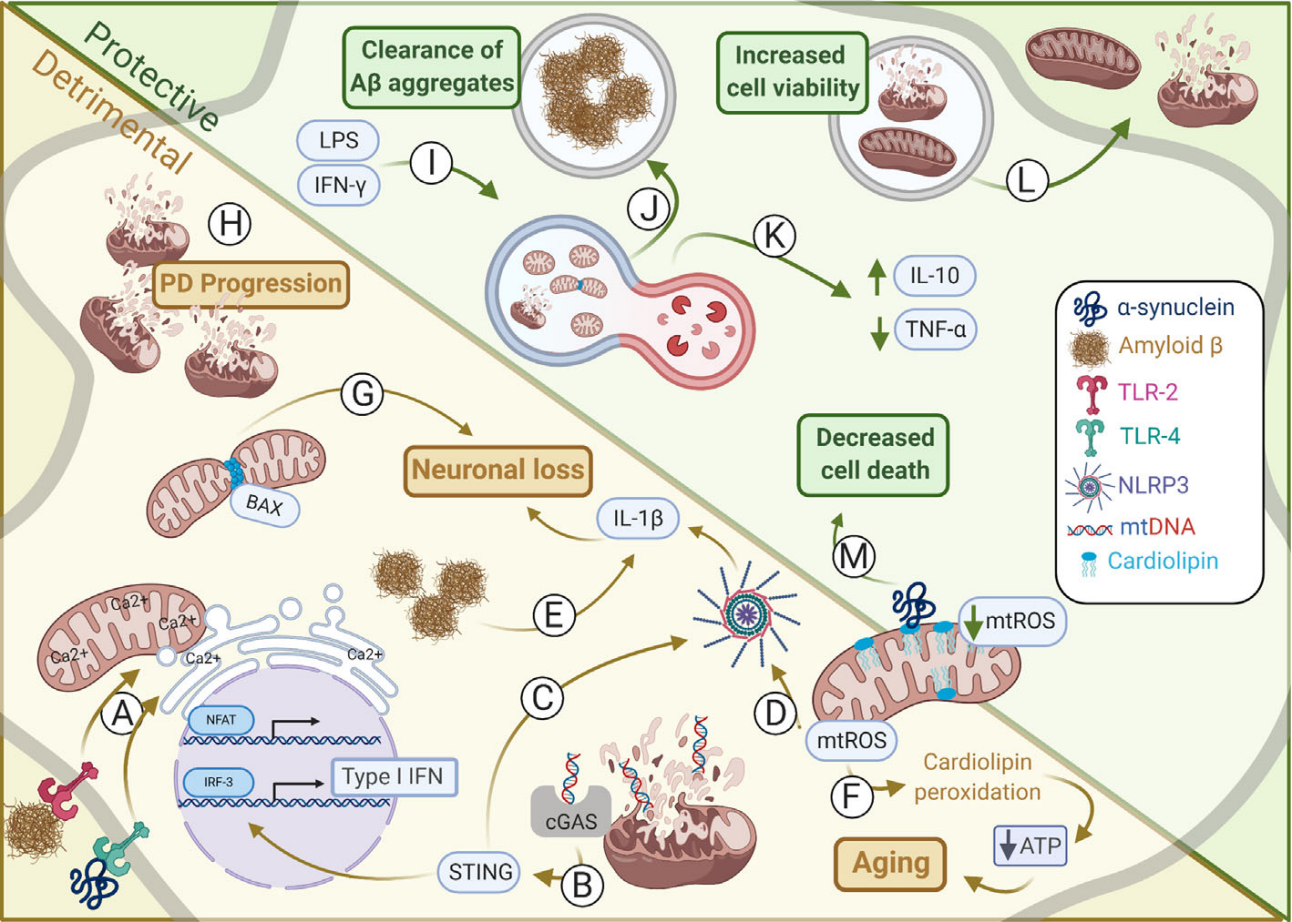
Mitochondrial alterations in protective and detrimental processes within CNS. Alterations in mitochondrial dynamics may induce either harmful or helpful immune responses affecting in CNS homeostasis. **(A)** Amyloid-β and α-synuclein induces TLR-2 and TLR-4 activation, respectively, promoting the interaction between outer mitochondria membrane (OMM) and endoplasmic reticulum (ER) membrane to synergically increase Ca^2+^ uptake and NFAT activation. **(B)** Type I IFN production is induced by mtDNA activation of cGAS-STING during sustained mitochondrial damage promoting neurodegeneration. **(C)** STING may also induce activation of NLRP3. **(D)** The NLRP3 may also be induced by mtROS thus coordinating IL-1β secretion by microglia and astrocytes promoting neuronal loss. **(E)** Amyloid-β aggregates induce NLRP3 inflammasome activation and IL-1β secretion by microglia. **(F)** mtROS induces cardiolipin peroxidation that deregulates ATP production, as observed during aging. **(G)** In neurons, BAX interacts with DRP1 inducing mitochondrial fragmentation. This is critical for BAX-dependent pore formation and neuronal survival. **(H)** Failure in mitophagy culminates in damaged mitochondria accumulation which contributes for Parkinson’s disease (PD) progress. **(I)** Mitophagy may be induced by IFN-γ and LPS upregulation of DRP1 and LC3, an autophagy-related protein. This is essential to restore tubular mitochondrial networks after inflammatory stimulation in astrocytes. **(J)** Microglial cells under mitophagy have elevated levels of intracellular Aβ aggregates, suggesting increased phagocytic activity, and thus clearing the harmful Aβ deposits. **(K)** PINK1 regulation of mitophagy is essential for CNS homeostasis establishment and induction of IL-10 and reduction of TNF-α secretion. **(L)** Instead of mitophagy the release of damage mitochondria may also minimize overall cell injury. Conversely, healthy mitochondria may also be donated from astrocytes to damaged neighboring neurons increasing its viability and maintaining its metabolism. **(M)** Cardiolipin can interact with α-synuclein preventing its aggregation by modifying its structure and impairing the release of cytosolic cytochrome c and thus inhibiting apoptosis and dampening cellular oxidative stress. Illustration prepared by the authors using www.biorender.com.

Although first believed to be solely the power houses of the cell, it is now accepted that mitochondria have an active role in many cellular processes, especially during inflammation. In **Figure 1** we summarize some of these relevant aspects concerning the correlation between mitochondria and brain disease. Although many of these diseases have their pathology linked to either mild or robust inflammatory responses, recent findings have unraveled that many of these mechanisms correlate with mitochondrial unbalanced function. Either because neuroinflammation can drastically impact cellular metabolism and further mitochondrial biology, for instance promoting mROS secretion or impairing ETC function, or because mitochondrial disfunction leads to the release of pro-inflammatory factors, as mtDNA. In this sense, innate immunity receptors as NLRP3 and MAVS greatly evidences the role of mitochondria as both effectors and sensors of neuroinflammation, respectively. Interestingly, structural changes in mitochondrial shape, size and turnover inside the cell, has also shown great relevance. For instance, OPA and MFN proteins defects, responsible for mitochondrial fusion are observed in Charcot-Marie-Tooth disease and optic atrophy. Mitochondria may also correlate with homeostasis and resolution of neuroinflammation. During mitophagy, for instance, there is the induction of IL-10 secretion and inflammation control, as observed in PD models. More interesting, inflammation activated astrocytes may actively transfer mitochondria to nearby neurons as an effort to avoid or reduce tissue damage, whereas damaged mitochondria are also released in order to avoid excessive ROS production, as described during stroke. In summary, the role of mitochondria during neuroinflammation and neurodegeneration has started to be better understood, not only unraveling important biological processes but also indicating that mitochondria-related immunometabolic pathways may serve as promising therapeutic targets for CNS diseases. This is corroborated by the fact that are currently 160 studies registered in www.clinicaltrials.gov found for the terms mitochondria and brain.

## Author Contributions

LGO and YSA wrote the manuscript and designed the figure; AHI wrote and edited the manuscript; JPSP wrote, elaborated the topics, edited and reviewed the manuscript. All authors contributed to the article and approved the submitted version.

## Funding

Neuroimmune interactions laboratory is supported by FAPESP (#2017/26170; #2017/22504-1), CAPES and CNPq.

## Conflict of Interest

The authors declare that the research was conducted in the absence of any commercial or financial relationships that could be construed as a potential conflict of interest.
